# Supplementary material for: Shifts in the conflict-coexistence continuum: Exploring social-ecological determinants of human-elephant interactions
Source: PLoS One. 2023 Mar 28;18(3):e0274155. doi: 10.1371/journal.pone.0274155 (PMC10047539; doi:10.1371/journal.pone.0274155)
Supplement: S1 File — (DOCX) [file pone.0274155.s002.docx]

**S2 Doc. Discussion guide**

1. **Focus group discussion guide.**
2. What are the main livelihood challenges faced by the community of this village?
3. What challenges are faced in agricultural practices (both farming and livestock keeping)?
4. Are there animals responsible for crop loss? If yes, which ones?
5. How much crop loss is due to elephants?
6. What time of the year do you normally encounter elephants? What do you think are the main reasons?
7. What mitigation measures are used to address HEC?
8. What measures do you consider most effective in mitigating HEC?
9. What do you consider a permanent solution for mitigating HEC? What are the challenges in implementing the solution?
10. Who do you think is responsible for mitigating HEC?
11. Is it important to have elephants in this area?
12. Do you think HEC has changed over the last 30 years? If so, how and why?
13. Other than elephant behavior, what other factors do you think cause HEC?
14. Historically, where do elephants pass in this landscape? are elephant paths within this village? Have their paths changed over time?
15. Any awareness of elephants being killed as a result of damage they caused?
16. Are there any other types of conflicts in this village?

**B. Key informant interview**

**a) *District Game Officer:***

1. What main challenges you are facing on conservation?
2. When did HEC become more common in the district?
3. What you think are the causes of HEC?
4. What do you think is the overall tolerance of the community in sharing the landscape with elephants?
5. What is the level of community participation in elephant conservation?
6. What specific benefits do people of Kilombero district gain from elephants?
7. Are there any compensation programs after elephant-induced damages?
8. Are there elephant specific attributes that have changed overtime? For example, their habituation to mitigation measures, change in food preference, changes in their habitats etc., which have contributed to HEC?
9. What do you think is the future of HEC if things remain the way they are currently?
10. What is the best way to address HEC?
11. What are the challenges you face at institutional level (as TAWA) in ensuring peaceful coexistence between people and elephants?

***b) Land Use Planning Officer:***

1. How serious is land related conflicts in the district?
2. Do you think HEC is a problem in this district?
3. What do you think are the main causes of HEC?
4. How is land availability in the district affecting human-elephants interaction?
5. Have large scale agricultural investments on land contributed to HEC? If yes how?
6. What do you think is the best way to address HEC?

***c)Agricultural Officer:***

1. Do you think HEC is a problem to agricultural practices in this district? If so, what is the estimate of crops lost to elephants per year (or during the past 1 year)?
2. What do you think are the main causes of HEC?
3. What is the food security situation of Morogoro rural district and how has it been affected by HEC? (Ask for specific statistics here e.g how much of food is lost to elephants and other animals districtwide, and for Kisemo, Mgude and Kidugalo villages)
4. What influences livelihood vulnerability in this district (i.e what are things that could potentially affect local communities’ livelihoods, especially for farmers?
5. What do you think is the future of HEC if things remain the way they are currently?
6. How is land availability in the district affecting human-elephants interaction?
7. How serious is land related conflicts in the district?
8. Are there any compensation programs after elephant-induced damages? If yes, how much?
9. What do you think is the best way to address HEC?

***d) Community Development:***

1. What are the main development challenges facing Morogoro rural district community?
2. Do you consider elephants as problems to people’s development?
3. What do you think are the main causes of HEC?
4. When did HEC become more common in this district?
5. Are there any community groups that support farmers in the districts (e.g VICOBA)? How are they helping farmers to overcome challenges of HEC?
6. What do you think is the future of HEC if things remain the way they are currently?
7. What do you think is the best way to address HEC?
