# Supplementary material for: Shifts in the conflict-coexistence continuum: Exploring social-ecological determinants of human-elephant interactions
Source: PLoS One. 2023 Mar 28;18(3):e0274155. doi: 10.1371/journal.pone.0274155 (PMC10047539; doi:10.1371/journal.pone.0274155)
Supplement: S1 Table — (DOCX) [file pone.0274155.s003.docx]

**S1 Table. Total population and sample sizes from each of the selected village in the study area.**

| **District** | **Village** | **Total Population** | **Total number of households** | **5% of Households** | **Actual number of surveyed households** |
| --- | --- | --- | --- | --- | --- |
| Morogoro Rural | Kidugalo | 3539 | 707 | 36 | 51 |
|  | Mgude | 2951 | 590 | 30 | 51 |
|  | Kisemo | 1800 | 360 | 18 | 50 |
| Sub-total |  | **8290** | **1657** | **96** | **152** |
|  |  |  |  |  |  |
| Kilombero | Kanyenja | 1700 | 284 | 15 | 26 |
|  | Katurukila | 4200 | 700 | 35 | 26 |
|  | Magombera | 996 | 166 | 9 | 50 |
|  | Mang’ula B | 3037 | 506 | 26 | 50 |
| Sub-total |  | **9933** | **1656** | **95** | **152** |
|  |  |  |  |  |  |
| Mvomero | Melela | 3246 | 541 | 28 | 50 |
|  | Mingo | 2709 | 452 | 23 | 50 |
|  | Lubungo | 2936 | 323 | 25 | 50 |
| Sub-total |  | **8891** | **1,316** | **90** | **150** |
| **TOTAL** |  | **27,114** | **4629** | **281** | **454** |
