# Supplementary figures and images for: Shifts in the conflict-coexistence continuum: Exploring social-ecological determinants of human-elephant interactions
Source: PLoS One. 2023 Mar 28;18(3):e0274155. doi: 10.1371/journal.pone.0274155 (PMC10047539; doi:10.1371/journal.pone.0274155)

**S3 Table. IRB Clearance**


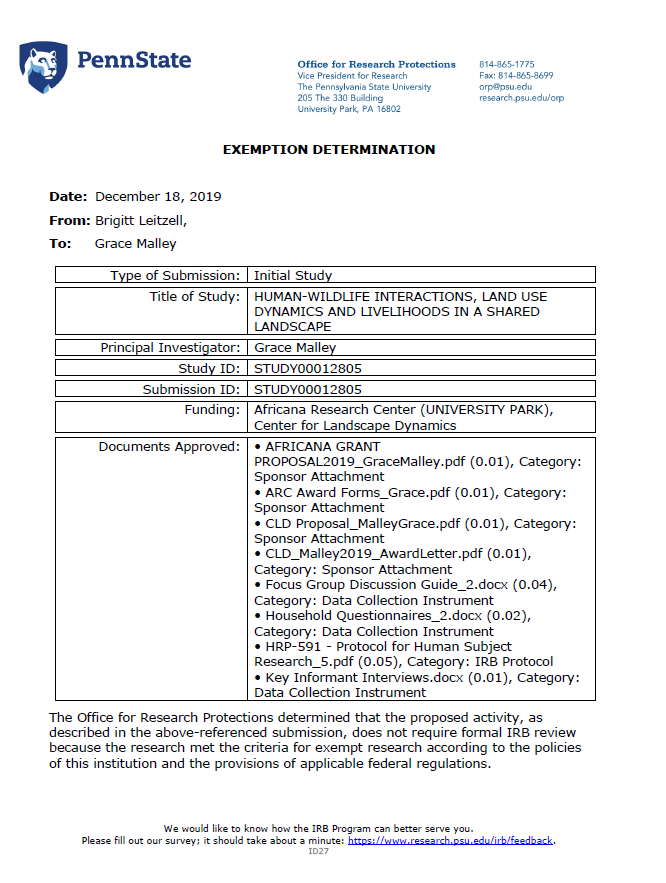

Supplement: S2 Table — (DOCX) [file pone.0274155.s004.docx]

**S8 Table. Variables included in the binary logistic regression analysis.**


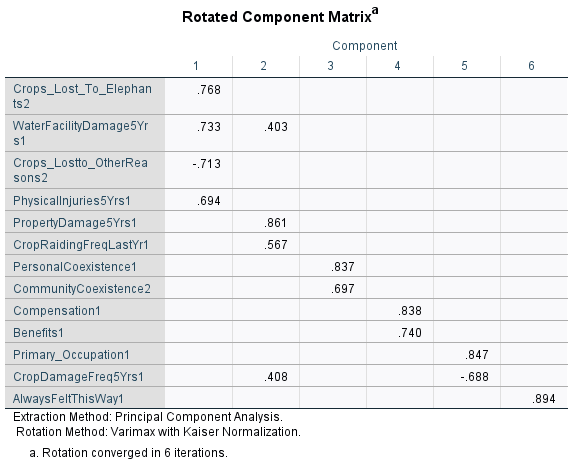

Supplement: S7 Table — (DOCX) [file pone.0274155.s009.docx]
