# Supplementary material for: Shifts in the conflict-coexistence continuum: Exploring social-ecological determinants of human-elephant interactions
Source: PLoS One. 2023 Mar 28;18(3):e0274155. doi: 10.1371/journal.pone.0274155 (PMC10047539; doi:10.1371/journal.pone.0274155)
Supplement: S3 Table — (DOCX) [file pone.0274155.s005.docx]

**S4 Table. The number of community members who participated in focus group discussion in each village and district.**

| **District** | **Village** | **Total number of members in Focus Group Discussions** | **Expert Interviews** |
| --- | --- | --- | --- |
| Morogoro Rural | Kidugalo | 20 | 4 |
|  | Mgude | 19 |  |
|  | Kisemo | 16 |  |
|  |  |  |  |
| Kilombero | Kanyenja | 15 | 4 |
|  | Katurukila | 15 |  |
|  | Magombera | 20 |  |
|  | Mang’ula B | 20 |  |
|  |  |  |  |
|  | Melela | 17 | 4 |
| Mvomero | Mingo | 20 |  |
|  | Lubungo | 18 |  |
|  |  |  |  |
| Sub-Total |  | 180 | 12 |
| **TOTAL** | | **192** | |
