# Supplementary material for: Shifts in the conflict-coexistence continuum: Exploring social-ecological determinants of human-elephant interactions
Source: PLoS One. 2023 Mar 28;18(3):e0274155. doi: 10.1371/journal.pone.0274155 (PMC10047539; doi:10.1371/journal.pone.0274155)
Supplement: S4 Table — (DOCX) [file pone.0274155.s006.docx]

**S5 Table. Household questionnaire**

|  |
| --- |
| **HUMAN-WILDLIFE INTERACTIONS, LAND USE DYNAMICS AND LIVELIHOODS IN A HETEROGENOUS LANDSCAPE**Principle Investigator: *Grace Malley* Institution: *Pennsylvania State University* *A: DEMOGRAPHIC INFORMATION* |
| 1. Respondent's gender |
| 1. What is your age |
| 1. What is your level of education |
| 1. What is your ethnic group? |
| 1. How many members are in your household? |
| 1. Where were you born? |
| 1. How long have you lived in this village? |
| 1. What is the name of your village? |
| 1. What is the primary livelihood activity of your household? |
| 1. Other than that (above), what other livelihood activities do you do? |
| 1. What is your gross monthly income (in TZS)? |
| 1. Do you own or rent land? |
| 1. How far is it located from your home? (Record the biggest, main land) |
| 1. What is the size of your land? |
| 1. What type of crops do you usually plant? |
| 1. Are these crops palatable to elephants? |
| 1. How many cropping cycles do you have in a year? |
| 1. If elephants visit your farm, do they normally go to the same field? |
| 1. Do you always have the same type of crops on these fields? |
| 1. Do you have livestock? If yes, how many? |
| 1. Are you or any member of this household a member of a VICOBA? If yes which one (s)? |
| 1. *HOW ARE LOCAL SUBSISTENCE FARMERS' LIVELIHOOD AFFECTED BY HUMAN-ELEPHANT INTERACTIONS* |
|  |
| 1. What main obstacles do you face to improve your life? |
| 1. What are the top three problems facing livestock keeping? |
| 1. What animals cause damage to your crops/livestock? |
| 1. How much of a problem are elephants? |
| 1. If elephants are a problem, why? |
| 1. Have you or anyone in your household experienced any of the following threats from elephants in the past 5 years? |
| 1. How many times did you experience crop damage in the past 5 years? |
| 1. How many times did you experience property damage in the past 5 years? |
| 1. How many times did you experience water facility damage in the past 5 years? |
| 1. How many times did you experience injuries in the past 5 years? |
| 1. How many times did you experience human death in the past 5 years? |
| 1. How many times did you experience damage to livestock in the past 5 years? |
| 1. Why do you think elephants attack people? |
| 1. How often did elephants raid your crops during the past year? |
| 1. How much of your harvest did you lose to elephants last year? (Show visual guide to help quantify) |
| 1. How much of your entire crops did you lose due to other reasons? |
| 1. At what time of the year does damage from elephants occur? |
| 1. What time of the day does elephant-caused damage occur? |
| 1. What actions do you take currently to prevent elephants damage? |
| 1. Over the last two years, did you or a member of your household gain any benefit from living near wild elephants? |
| (Follow up on Qn.20): If yes, please specify what kind of benefit |
| 1. Have you received any compensation after elephant damage? |
| If yes, in what form? |
| 1. What do you think should be done to address elephant problem? |
| 1. How often do you see elephants outside the protected area? |
| *C: LOCAL PEOPLE’S ATTITUDES TOWARDS ELEPHANTS* |
|  |
| 1. In your opinion, how do people and elephants live together in this region? |
| 1. Which statement describes your attitude toward elephants most accurate? |
| 1. In a scale of 1-5 (1=I like elephants very much and 5=I hate elephants), where would you place yourself? |
| 1. (Follow up for Qn.3): Have you always felt this way towards elephants? |
| 1. (Follow up on Qn.3): If you have not always felt this way, when did your feelings towards elephants change? |
| 1. Since when do you think human-elephant conflict has become more severe? |
| 1. What do you think are the main causes of human-elephant conflicts? |
| 1. Why do you think elephants attack people? |
| 1. Who should be responsible for mitigating HEC? |
| 1. Are you aware of any efforts to conserve elephants? |
| If yes please specify which one |
| 1. Do you or a member of your household participate in elephant conservation? |
| 1. Are you participating in collective HEC mitigation efforts? |
| 1. (Follow up on Qn.35) If you are participating, how? |
| 1. Is it important for this protected area (specify the name of the nearby protected area) to have wild elephants in the future? |
| 1. What do you think is the trend of HEC in the past 30 years? |
| 1. In your opinion, how do you see the relationship between humans and elephants in the future? |
