# Supplementary material for: Shifts in the conflict-coexistence continuum: Exploring social-ecological determinants of human-elephant interactions
Source: PLoS One. 2023 Mar 28;18(3):e0274155. doi: 10.1371/journal.pone.0274155 (PMC10047539; doi:10.1371/journal.pone.0274155)
Supplement: S5 Table — (DOCX) [file pone.0274155.s007.docx]

**S6 Table:** **Summary of household survey for the study area and for each district.**

| **S/N** | **Variable** | **Entire Study Area** | **Mvomero** | **Kilombero** | **Morogoro rural** |
| --- | --- | --- | --- | --- | --- |
| 1 | Gender | 55.9% women, 44.1% men | 60% women, 40% men | 55%women, 45% men | 52% women, 48% men |
| 2 | Median age group | 45-54 | 45-54 | 45-54 | 45-54 |
| 3 | Level of Education | 93.5% no formal education or only primary school | 93.3% no formal education or only primary school | 94.7% no formal education or only primary school | 92.7% no formal education or only primary school |
| 4 | Household size | 5 | 5 | 5 | 5 |
| 5 | Number of years lived in the village (Median) | 28 | 32.5 | 16 | 31.5 |
| 6 | Other livelihood activities (apart from agriculture) | 98.2 % No | 99.1% No | 96.3% No | 98.7% No |
| 7 | Income (Monthly) | 90.8% earned 200,000TZS or less (less than $100) | 89.3% earned 200,000TZS or less (less than US$100) | 94% earned 200,000TZS or less (less than US $100) | 88.8% earned 200,000TZS or less (less than US$100) |
| 8 | Land ownership | 99.6% Yes | 99.3 Yes | 100% Yes | 99.9% Yes |
| 9 | Farm size | Average 2.9 acres per household. | Average 3.38 acres per household. | Average 2.78 acres per household. | Average 2.5 acres per household. |
| 10 | Number of cropping cycles | 51.1% one; 47.8% two | 71.3% one, 26.7 two | 64% one, 36% two | 82.2% two; 17.2% one |
| 11 | Elephants raid same field? | Yes, most of the time | Yes, most of the time | Yes, most of the time | Yes, most of the time |
| 12 | How much of a problem are elephants? | 92.7% major | 98.7% major | 93.4% major | 86.2% major |
| 13 | Crop damage frequency in the past 5 years | 93% more than 10 times | 92.7% more than 10 times | 89.3% at least 10 times | 82.2% more than 10 times |
| 14 | Property damage frequency in the past 5 years | 80% | 71.3% none | 78% none | 90.8% none |
| 15 | Water facility damage frequency in the past 5 years | 83.9% none | 55% none | 99.3% none | 97.4% none |
| 16 | Physical injuries frequency in the past 5 years | 96.2%% none | 90% none | 100% none | 98.7% none |
| 17 | Human death experienced in the past 5 years | 98.5% none | 96.3% none | 100% none | 99.3% none |
| 18 | Livestock damage frequency in the past 5 years | 92.1% none | 94.7% none | 99.3% none | 82.2% none |
| 19 | Crop raiding frequency experienced last year | 100% yes; 64.7% at least 3 times | 100% yes; 84.6% at least 3 times | 100% yes; 63.9% at least 3 times | 100% yes; 46.1% at least 3 times |
| 20 | Amount of crops lost to elephants last year | 63.9% lost at least 50% of entire crops | 93.3% lost at least 50% of entire crops | 47.4% lost at least 50% of entire crops | 47.3% lost at least 50% of entire crops |
| 21 | Amount of crops lost to other reasons last year | 33.7% none | 54% lost at least 50% of their crops to other reasons | 96.7% none | 30.2% lost at least 50% of their crops to other reasons |
| 22 | HEC Trend in the past 5 years | 100% increased | 100% increased | 100% increased | 100% increased |
| 23 | Time of the year damage from elephants occur | 86% all year round | 94% all year round | 96.7% all year round | 67.8% all year round |
| 24 | Why HEC increased | 99% number of elephants has increased | 99.3% number of elephants has increased | 98% number of elephants has increased | 95% number of elephants has increased |
| 25 | Time of the day elephant-caused damage occur | 41.2% anytime | 39.3% anytime | (70.4%) anytime | 76.3% night |
| 26 | Benefits from elephants | 80.8% No | 84% No | 77.6% No | 90.8% No |
| 27 | Received compensation? | 83.7% No | 86% No | 97.4% No | 77.6% No |
| 28 | How often do you see elephants outside the protected area | 75.3% Almost everyday | 94.7% Almost everyday | 70.4% Almost everyday | 61.2% Almost everyday |
| 29 | How do people and elephants live together in this village | 89.4% Unpeacefully | 93% Unpeacefully | 97.4% Unpeacefully | 94.7% Unpeacefully |
| 30 | Personal tolerance to share space with elephants | 54.6% would prefer elephants to be eradicated | 75% would prefer elephants to be eradicated | 53.9% conditional tolerance | 64.5% would prefer elephants to be eradicated |
| 31 | In a scale of 1-5 (1=I like elephants very much and 5=I hate elephants), how would you define your feelings towards elephants? | 88.1% hate or dislike elephants | 98% dislike or hate elephants | 72.4% dislike or hate elephants | 92.1% dislike or hate elephants |
| 32 | Have you always felt this way towards elephants? | 84.1% No | 90.7% No | 77.3% No | 84.3% No |
| 33 | Why Feelings towards elephants changed | 81.7% Damage to crops | 86% Damage to crops | 77% Damage to crops | 81.6% Damage to crops |
| 34 | If not, when did your feelings towards elephants change? | 67% 1 to 5 years ago | 68.7% 1 to 5 years ago | 57.9% 1 to 5 years ago | 73% 5-6 years ago |
| 35 | When did human-elephant conflict become more severe? | 69.8% 1 to 5 years ago | 72% 1 to 5 years ago | 64.5% 1 to 5 years ago | 73% 1 to 5 years ago |
| 36 | Causes of human-elephant conflicts | 94.9% the number of elephants has increased | 99.3% the number of elephants has increased | 95.4% the number of elephants has increased | 90.1% the number of elephants has increased |
| 37 | Knowledge about conservation efforts present in the village | 54% Yes | 88% No | 53.9% Yes | 59.2% Yes |
| 38 | Participation in conservation | 86.6% No | 70% No | 84.9% No | 91.4% No |
| 39 | Human -elephant conflicts trend in the past 30 years | 96.9% Increasing | 100% Increasing | 97.4% Increasing | 94.1% Increasing |
| 40 | How do you see the relationship between humans and elephants in the future | 95.6% Worse | 99.3% Worse | 96.1% Worse | 91.4% Worse |
| 41 | Willingness to participate in conservation efforts | 90.5% Yes | 92% Yes | 90.1% Yes | 89.5% Yes |
