# Supplementary material for: Shifts in the conflict-coexistence continuum: Exploring social-ecological determinants of human-elephant interactions
Source: PLoS One. 2023 Mar 28;18(3):e0274155. doi: 10.1371/journal.pone.0274155 (PMC10047539; doi:10.1371/journal.pone.0274155)
Supplement: S6 Table — (DOCX) [file pone.0274155.s008.docx]

**S7 Table. Research permit**
