# Supplementary material for: Shifts in the conflict-coexistence continuum: Exploring social-ecological determinants of human-elephant interactions
Source: PLoS One. 2023 Mar 28;18(3):e0274155. doi: 10.1371/journal.pone.0274155 (PMC10047539; doi:10.1371/journal.pone.0274155)
Supplement: S8 Table — (DOCX) [file pone.0274155.s010.docx]

**S9 Table.** **LULC Classification Accuracy Assessment in percentage for 1994, 2007 and 2020 for Kilombero, Morogoro Rural and Mvomero districts.**
